# Phospholipase C and Diacylglycerol Mediate Olfactory Responses to Amino Acids in the Main Olfactory Epithelium of an Amphibian

**DOI:** 10.1371/journal.pone.0087721

**Published:** 2014-01-28

**Authors:** Alfredo Sansone, Thomas Hassenklöver, Adnan S. Syed, Sigrun I. Korsching, Ivan Manzini

**Affiliations:** 1 Institute of Neurophysiology and Cellular Biophysics, University of Göttingen, Göttingen, Germany; 2 Institute of Genetics, University of Cologne, Cologne, Germany; 3 Center for Nanoscale Microscopy and Molecular Physiology of the Brain (CNMPB), University of Göttingen, Göttingen, Germany; Monell Chemical Senses Center, United States of America

## Abstract

The semi-aquatic lifestyle of amphibians represents a unique opportunity to study the molecular driving forces involved in the transition of aquatic to terrestrial olfaction in vertebrates. Most amphibians have anatomically segregated main and vomeronasal olfactory systems, but at the cellular and molecular level the segregation differs from that found in mammals. We have recently shown that amino acid responses in the main olfactory epithelium (MOE) of larval *Xenopus laevis* segregate into a lateral and a medial processing stream, and that the former is part of a vomeronasal type 2 receptor expression zone in the MOE. We hypothesized that the lateral amino acid responses might be mediated via a vomeronasal-like transduction machinery. Here we report that amino acid-responsive receptor neurons in the lateral MOE employ a phospholipase C (PLC) and diacylglycerol-mediated transduction cascade that is independent of Ca^2+^ store depletion. Furthermore, we found that putative transient receptor potential (TRP) channel blockers inhibit most amino acid-evoked responses in the lateral MOE, suggesting that ion channels belonging to the TRP family may be involved in the signaling pathway. Our data show, for the first time, a widespread PLC- and diacylglycerol-dependent transduction cascade in the MOE of a vertebrate already possessing a vomeronasal organ.

## Introduction

The peripheral olfactory system of vertebrates consists of neuroepithelia specialized for odor detection [1]. Teleost fishes possess a single olfactory sensory surface harboring ciliated and microvillous olfactory receptor neurons (ORNs; [2]). Anatomically segregated olfactory subsystems first appeared in amphibians ([2]; but see [3]). Most amphibians possess a vomeronasal organ (VNO) with microvillous receptor neurons, but many species, including *Xenopus laevis*, still have a ‘fish-like’ main olfactory epithelium (MOE), containing both ciliated and microvillous neurons [4]. Fully terrestrial vertebrates typically possess anatomically segregated olfactory subsystems with ciliated ORNs located in the MOE, septal organ and the Grueneberg ganglion, and microvillous receptor neurons almost exclusively present in the VNO [1].

In the single sensory surface of teleost fishes and the VNO of rodents microvillous receptor neurons express vomeronasal type 1 and type 2 receptors, Gα_i_ or Gα_o_, and generally signal via a phospholipase C (PLC)-mediated pathway that leads to opening of the canonical transient receptor potential channel 2 (TRPC2). Ciliated receptor neurons express OR-type olfactory receptors and Gα_olf_, and typically signal via the canonical cAMP pathway [1], [5]. In amphibians, microvillous and ciliated ORNs of the MOE segregate into a lateral and a medial odor-processing stream, respectively. We showed recently that the medial stream exhibits the canonical cAMP-mediated transduction pathway [6], whereas the lateral stream contains ORNs expressing *v2r* genes and Gα_i_ or Gα_o_. However, the further transduction pathway in the lateral stream is not known. Amino acids are a main odor class for aquatic vertebrates, and elicit strong responses in both streams [6].

Here we investigated the transduction mechanism of the amino acid-sensitive ORN subpopulation of the lateral MOE of larval *Xenopus laevis*, and show that these neurons possess a PLC and diacylglycerol (DAG)-mediated transduction pathway that is independent of intracellular Ca^2+^ store depletion. We also show that two putative transient receptor potential (TRP) channel blockers significantly reduce the amplitude of amino acid-induced responses in these ORNs, consistent with an involvement of TRP channels. Taken together, these data suggest that the MOE of *Xenopus* not only expresses vomeronasal receptors [7], but also the transduction machinery associated with vomeronasal receptors in the mammalian system.

## Methods

### Ethics Statement

All experiments are approved by the Göttingen University Committee for Ethics in Animal Experimentation.

### Preparation of acute slices of the MOE

Larvae of *Xenopus laevis* (of either sex, stages 50 to 54; see [8]) were cooled in iced water to produce complete immobility and killed by transection of the brain at its transition to the spinal cord. A block of tissue containing the MOE, the olfactory nerves and the forebrain was cut out. The tissue block was glued onto the stage of a vibroslicer (VT 1200 s, Leica, Bensheim, Germany), covered with bath solution (see below) and sliced horizontally into 130–150 µm thick slices.

### Solutions, staining protocol and stimulus application

Bath solution consisted of (in mM): 98 NaCl, 2 KCl, 1 CaCl_2_, 2 MgCl_2_, 5 glucose, 5 Na-pyruvate, 10 HEPES. The composition of the Ca^2+^-free bath solution was (in mM): 98 NaCl, 2 KCl, 2 MgCl_2_, 5 glucose, 5 Na-pyruvate, 10 HEPES, 2 EGTA. All bath solutions were adjusted to pH 7.8 and had an osmolarity of 230 mOsmol/l. Tissue slices (see above) were transferred to a recording chamber, and 200 µl of bath solution containing 50 µM Fluo-4/AM (Molecular Probes, Leiden, The Netherlands) was added. Fluo-4/AM was dissolved in DMSO (Sigma) and Pluronic F-127 (Molecular Probes). The final concentrations of DMSO and Pluronic F-127 did not exceed 0.5% and 0.1%, respectively. Cells of the MOE of larval *Xenopus laevis* express multidrug resistance transporters with a wide substrate spectrum, including Ca^2+^-indicator dyes [9]. To avoid transporter-mediated destaining of the slices, 50 µM MK571 (Alexis Biochemicals, Grünberg, Germany), an inhibitor of multidrug transporters, was added to the incubation solution. The preparations were incubated on a shaker at room temperature for 35 minutes. The slices were then fixed in a recording chamber, which was constantly perfused with bath solution applied by gravity feed from a storage syringe through a funnel applicator placed directly above the MOE. The stimuli were applied into the funnel without stopping the flow. Bath solution was constantly removed from the recording chamber through a syringe needle. All experiments were conducted at room temperature. The reproducibility of the responses was verified by regularly repeating the application at least twice. The minimum interstimulus interval was at least two minutes in all of the experiments. As odors, we used 19 amino acids, and a mixture of alcohols, aldehydes and ketones (for a detailed list of the odorants see [6]) all purchased from Sigma (Deisenhofen, Germany). The odorants were dissolved in bath solution (10 mM stock) and applied at a final concentration of 100 µM for each component in the mixtures. Forskolin, cyclopiazonic acid (CPA), 2-aminoethoxydiphenyl borate (2-APB), and arachidonic acid were purchased from Sigma. U-73122, U-73343, Xestospongin C and 1-Stearoyl-2-arachidonoyl-sn-glycerol (SAG) were from Calbiochem (Darmstadt, Germany), and SKF-96365 from Abcam (Cambridge, United Kingdom). All pharmacologic agents were dissolved in concentrated stock solutions, aliquoted and frozen. Aliquots were thawed only once and the working solutions (see specific experiments) were freshly prepared before performing the experiment. SAG was applied at concentrations of 200 and 500 µM. For all other chemicals the used concentrations are given in the Figure legends.

### Ca^2+^ imaging and data evaluation

Changes of intracellular calcium concentrations of individual ORNs were monitored using a laser-scanning confocal microscope (LSM 510/Axiovert 100 M, Zeiss, Jena, Germany). Fluorescence images (excitation at 488 nm; emission >505 nm) of the MOE were acquired at 1 Hz, with 10 images taken as control before the onset of stimulus delivery. The thickness of the optical slices excluded fluorescence detection from more than one cell layer. The data were analyzed using custom written programs in MATLAB (Mathworks, Natick, USA). To facilitate selection of regions of interest, a ‘pixel correlation map’ was obtained by calculating the cross-correlation between the fluorescence signals of a pixel to that of its immediate neighbours and then displaying the resulting value as a grayscale map. As physiological responses often give similar signals in adjacent pixels, this method specifically highlights those pixels. In contrast, pixels that contain only noise show uncorrelated traces and thus appear dark in the cross-correlation map [10]. Fluorescence changes for individual regions of interest, i.e. individual ORNs, are given as ΔF/F values. The fluorescence changes ΔF/F were calculated as ΔF/F = (F−F_0_)/F_0_, where F was the fluorescence averaged over the pixels of an ORN, while F_0_ was the average fluorescence of that ORN prior to stimulus application, averaged over three images [11]. A response was assumed if the following criteria were met: (i) the maximum amplitude of the calcium transient had to be higher than the maximum of the prestimulus intensities; (ii) the onset of the response had to be within ten frames after stimulus application. A blocker/inhibitor was assumed to affect a response if the following criteria were met: (i) the maximum amplitude of the calcium transient had to be reduced by at least 30% with the blocker/inhibitor added; (ii) the response had to recover during the washout of the blocker/inhibitor. Averaged data are presented as mean ± standard error of the mean (SEM). Statistical significance was determined by paired Student's t-test or Chi-squared test.

## Results

### All responses to amino acid odors are driven by extracellular Ca^2+^ and are independent of store depletion

**Figure 1:**
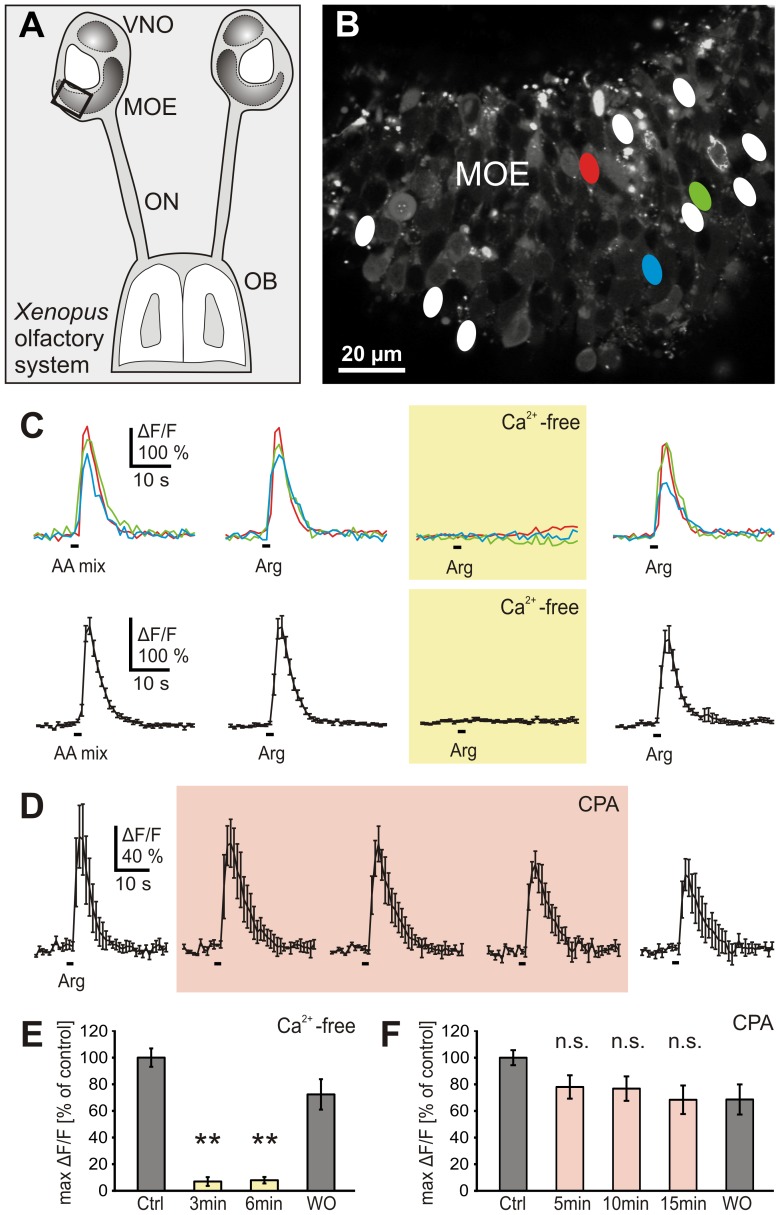
Amino acid-induced Ca^2+^ responses depend on extracellular Ca^2+^ and are independent of store depletion. *A*, schematic representation of the olfactory system of larval *Xenopus laevis*. The black rectangle indicates the approximate epithelial region of the slice shown in *B*. *B*, Fluo-4 stained acute slice of the MOE. The ovals represent all L-arginine-sensitive ORNs of this slice. *C*, time courses of intracellular Ca^2+^ transients of three amino acid-responsive ORNs (colored traces, see ovals in *B*) stimulated with a mixture of 19 amino acids (100 µM each) or L-arginine (100 µM) in standard and Ca^2+^-free bath solution. The L-arginine responses were abolished in Ca^2+^-free solution (yellow-shaded panel; application for 3 min) and recovered in standard bath solution. Mean Ca^2+^ transients ± SEM of all L-arginine-sensitive ORNs of the slice in *B* (11 ORNs) are shown in the lower part (black traces). *D*, depletion of intracellular Ca^2+^ stores via application of CPA (20 µM; light-red-shaded panel) did not affect L-arginine-evoked Ca^2+^ responses (shown as mean ± SEM of all three responsive ORNs of another acute MOE slice. *E*, mean responses (±SEM), expressed as percentage of control response to L-arginine, of 33 ORNs (11 slices) in standard (dark grey columns) and Ca^2+^-free bath solution (yellow columns; respectively 3 and 6 min after application of Ca^2+^-free bath solution). The responses were virtually abolished in Ca^2+^-free bath solution (**p<0.01; paired Student's t-test). *F*, mean responses (±SEM), expressed as percentage of control response to L-arginine, of 28 ORNs (6 slices) in standard (dark grey columns) and bath solution with 20 µM CPA (light-red columns; respectively 5, 10 and 15 min after application of CPA). Washout time was 5 min. No significant reduction of the Ca^2+^ responses was observed (p>0.05; paired Student's t-test). [AA mix, amino acid mixture; Arg, L-arginine; Ctrl, control; n.s., non significant; OB, olfactory bulb; ON, olfactory nerve; WO, washout].

To identify amino acid-sensitive ORNs in acute slices of the MOE (Figure 1A, B), we first applied a mixture of 19 amino acids. The frequency of responsive ORNs and the observed amplitudes of amino acid induced Ca^2+^-transients were consistent with results obtained in previous work [12], [13]. In a second step, we searched for ORNs sensitive to L-arginine, an individual amino acid and thus a more specific stimulus. 63% of the ORNs sensitive to the mixture of 19 amino acids responded also to L-arginine (11 slices). Responses to L-arginine were completely eliminated in Ca^2+^-free solution, i.e. responses were dependent on extracellular calcium, and recovered completely after returning to standard conditions (Figure 1C). Identical results were obtained in all experiments (33 ORNs, 11 slices; Figure 1E). Responses to the mixture of 19 amino acids were also totally suppressed in Ca^2+^-free conditions (8 ORNs, 3 slices; data not shown), indicating that all amino acid-induced responses generally rely on extracellular Ca^2+^. Application of CPA, a drug that induces depletion of intracellular Ca^2+^ stores, did not significantly affect L-arginine-induced responses (28 ORNs, 6 slices; Figure 1D, F).

### PLC is required for nearly all amino acid responses in the lateral MOE, whereas in non-lateral regions PLC-independent amino acid responses predominate

**Figure 2:**
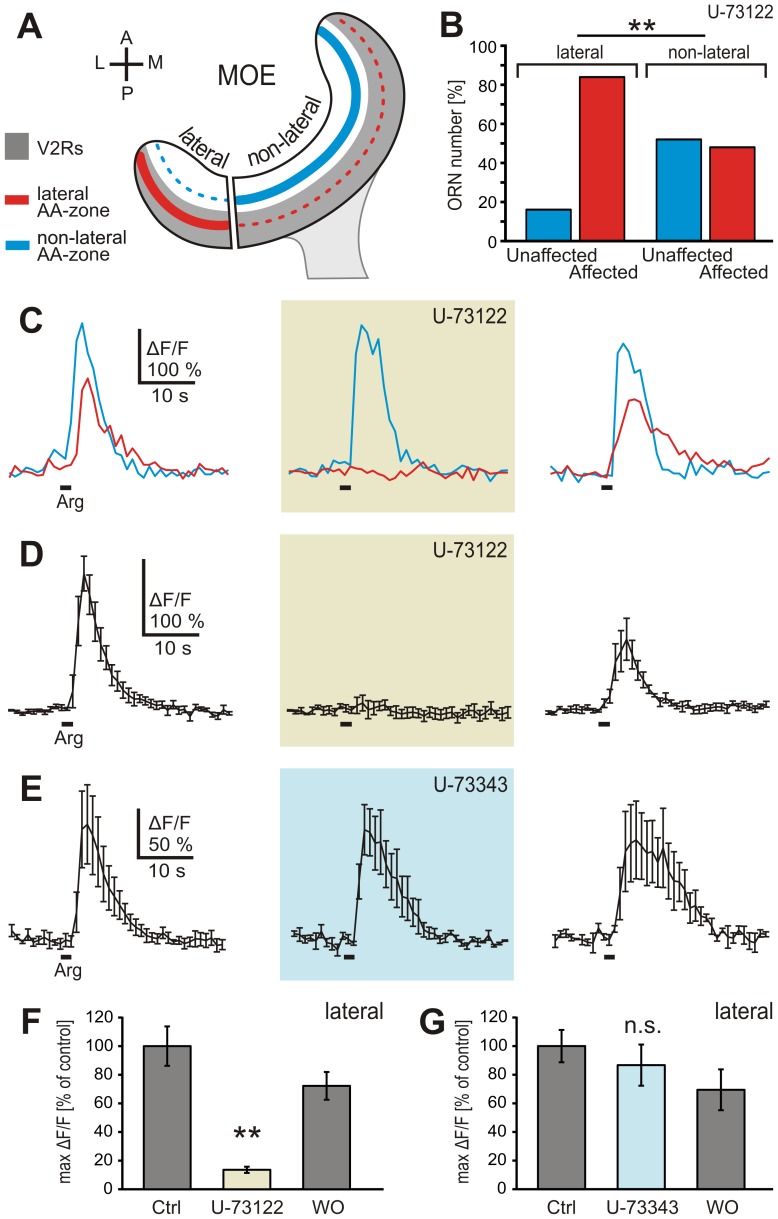
The PLC inhibitor U-73122 affects most of the L-arginine-induced Ca^2+^ responses in the lateral MOE. *A*, schematic representation indicating the approximate borders between the lateral and non-lateral part of the MOE of larval *Xenopus laevis*. Also given are the epithelial *v2r*-expression zone (grey band), and the location of the lateral (red line) and non-lateral (cyan line) amino acid-sensitive ORN subpopulations (see [7]). Note that the two subpopulations are not strictly confined to the lateral and non-lateral MOE, but rather gradually diminish from lateral to medial and medial to lateral epithelial positions (red and cyan dotted lines). *B*, relative number of ORNs unaffected and affected by U-73122 in the lateral (25 cells, 8 slices) and non-lateral (25 cells, 11 slices) part of the MOE, expressed as percentage of total ORNs tested. *C*, L-arginine-induced Ca^2+^ responses of two ORNs of an individual slice of the lateral MOE (cyan and red trace). Incubation with 10 µM U-73122 (application for 5 min; brown-shaded panel) led to a strong reduction of the Ca^2+^ transient in one of the ORNs (red trace), whereas the second ORN was not affected (cyan trace). The response of the affected ORN recovered after a washout time of 10 min (right-hand panel). *D*, mean Ca^2+^ transient (±SEM) of all five L-arginine-responsive ORNs of an acute slice preparation (lateral part of the MOE; all responsive ORNs of this slice were affected by U-73122). With 10 µM U-73122 in the bath solution (5 min; brown-shaded panel) the mean response is almost completely suppressed. After a washout time of 10 min the response recovered (right-hand panel). *E*, mean Ca^2+^ transient (±SEM) of all three L-arginine-responsive ORNs of another acute slice preparation (lateral part of the MOE) treated with U-73343 (10 µM; 5 min). The L-arginine response was not inhibited (light-blue-shaded panel). *F*, the average reduction of the Ca^2+^ transient of the ORN population affected by U-73122 was highly significant. *G*, the mean response amplitude (±SEM) of all ORNs tested (20 ORNs, 7 slices) was not significantly reduced by U-73343. Statistical analysis was performed using Chi-squared test (*B*, **p<0.01) and paired Student's t-test (*F*, **p<0.01; *G*, p>0.05). [AA-zone, amino acid-responsive zone; V2Rs, vomeronasal type 2 receptor expression zone; A, anterior; P, posterior; L, lateral; M, medial].

The PLC-inhibitor U-73122 has been shown to block urine-induced responses of vomeronasal receptor neurons (VRNs) in rodents [14]–[17]. Figure 2A depicts the vomeronasal type 2 receptor expression zone of the MOE (grey shaded band), and the epithelial location of the two main subpopulations of amino acid sensitive ORNs (red, lateral subpopulation; cyan, non-lateral subpopulation). U-73122 induced strong and reversible reduction of L-arginine-induced Ca^2+^ transients in 84% of the tested ORNs of the lateral MOE (21 out of 25 ORNs, 8 slices). The responses of the four remaining ORNs were not affected at all (Figure 2B, C). Figure 2D shows the mean intracellular Ca^2+^ transients of all L-arginine-sensitive ORNs of an individual MOE slice containing solely ORNs affected by the inhibitor. On average, the amplitude of L-arginine-induced Ca^2+^ responses was reduced by more than 85% under U-73122 (21 affected ORNs; Figure 2F). U-73343, an analogue of U-73122 with very weak activity, showed no significant effect on L-arginine-induced responses (20 ORNs, 7 slices; different set of slices; Figure 2E, G). In contrast, only 48% of the L-arginine-sensitive ORNs of the non-lateral MOE (see Figure 2A, B) were affected by U-73122 (12 out of 25 ORNs, 11 slices). The responses of 13 ORNs were not affected at all (Figure 2B). This distribution is significantly different from that observed in the lateral MOE, suggesting opposing lateral-to-medial gradients for PLC-dependent and PLC-independent amino acid-sensitive neurons.

**Figure 3:**
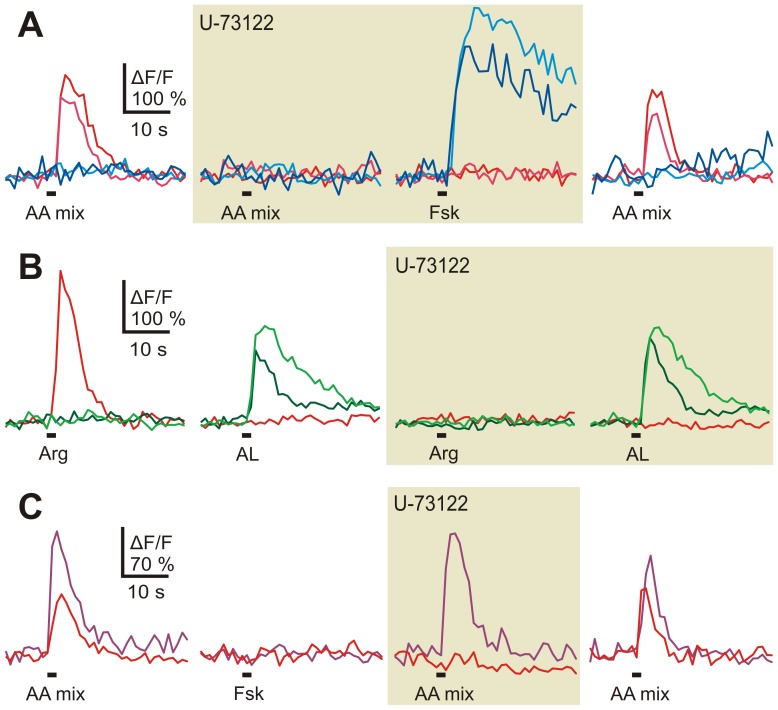
U-73122 specifically inhibits amino acid-sensitive ORNs. *A*, amino acid and forskolin-induced (50 µM) Ca^2+^ transients of four individual ORNs of an acute slice preparation of the lateral MOE (left-hand panel; red and magenta traces, amino acid sensitive ORNs; blue and cyan traces, forskolin-sensitive ORNs). U-73122 (10 µM; application for 5 min; brown-shaded panel) affected only the two amino acid-sensitive ORNs. After 10 min washout the response of the amino acid-sensitive ORNs recovered (right-hand traces). Similar results were obtained in all tested ORNs (42 cells, 3 slices). *B*, responses to L-arginine and alcohols, aldehydes and ketones of three individual ORNs of an acute slice preparation of the lateral MOE (left-hand panels; red trace, L-arginine-sensitive ORN; green traces, ORNs sensitive to alcohols, aldehydes and ketones). U-73122 (10 µM; 5 min; brown-shaded panel) did not affect Ca^2+^ transients induced by application of a mixture of alcohols, aldehydes and ketones. Note that the L-arginine-responsive ORN (red trace) was inhibited. Similar results were obtained in all tested ORNs (34 cells, 2 slices). *C*, two ORNs of an acute slice (lateral MOE) responded upon application of amino acids (red and purple traces). After application of U-73122 (10 µM; 5 min; brown-shaded panel) the response of one ORN (red trace) was suppressed, the response of the second ORN (purple trace) was unaffected. Both ORNs did not show sensitivity to forskolin (50 µM). After 10 min washout the response of the amino acid-sensitive ORN recovered almost completely (right-hand trace). Similar results were obtained in all four amino acid-sensitive ORNs that were unaffected by U-73122 (3 slices). [AL, mixture of alcohols, aldehydes and ketones; Fsk, forskolin].

As a control for the specificity of U-73122, we applied in a different set of experiments (3 slices) this inhibitor together with either the mixture of amino acids or forskolin (50 µM), an activator of the adenylate cyclase that is known to activate ORNs endowed with the canonical cAMP-mediated pathway [12]. While most of the lateral amino acid-induced responses (12 out of 16 ORNs) were suppressed, responses to forskolin (26 ORNs) persisted under U-73122 (Figure 3A; traces are from ORNs of the same slice). A similar experiment was performed with L-arginine and a mixture of alcohols aldehydes and ketones, known cAMP-transduced odors ([6]; 2 slices). While all L-arginine responses (7 ORNs) were suppressed, responses to alcohols, aldehydes, and ketones (27 ORNs) were not inhibited by U-73122 (Figure 3B). Interestingly, the amino acid-sensitive ORNs that were not affected by U-73122 did also not respond to forskolin (Figure 3C, also seen in other three ORNs, same dataset as in Figure 3A).

### Diacylglycerol induces a transient increase in intracellular Ca^2+^ in amino acid-sensitive ORNs in the lateral MOE

**Figure 4:**
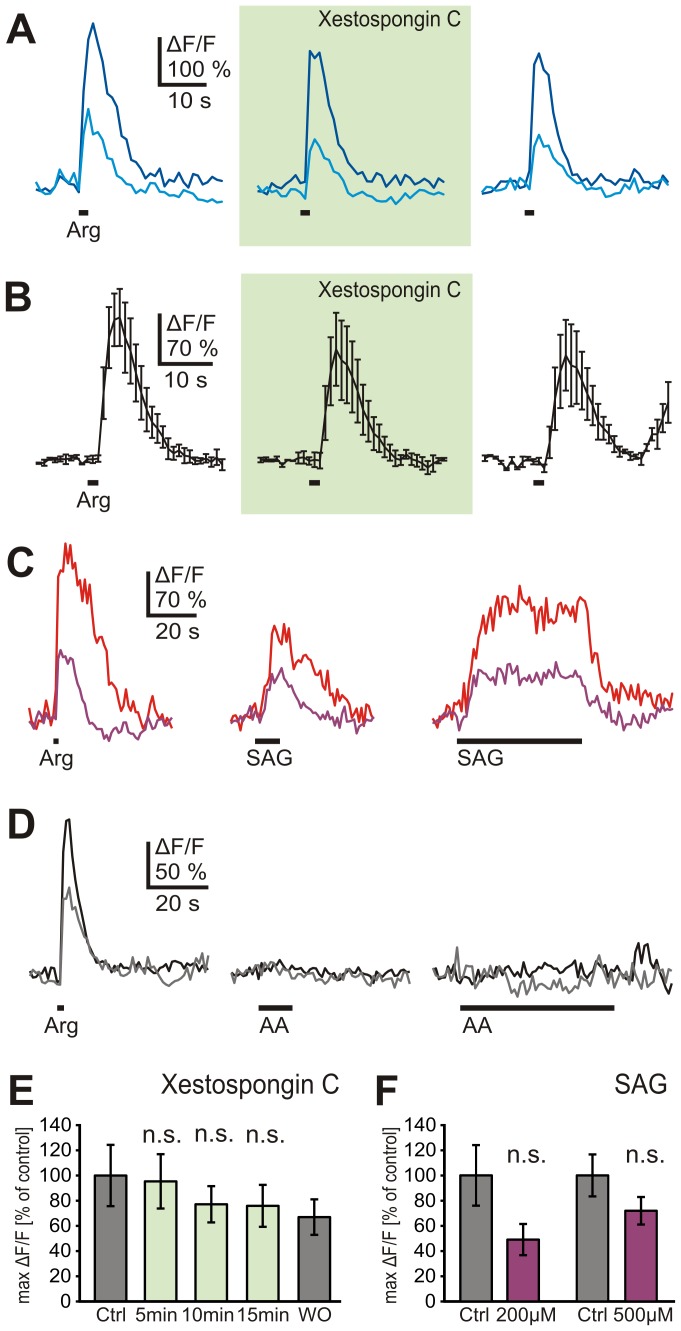
Diacylglycerol but not phosphatidylinositol-3-phosphate or arachidonic acid is involved in amino acid-signaling in the lateral MOE. *A*, L-arginine-induced Ca^2+^ responses of two ORNs of an individual slice of the lateral MOE (cyan and blue trace). Incubation with Xestospongin C (500 nM; application for 10 min; green-shaded panel) did not affect the responses. *B*, mean Ca^2+^ transients (±SEM) of all three L-arginine-responsive ORNs of an acute slice preparation (lateral MOE). The responses were not affected by Xestospongin C (500 nM; 10 min; green-shaded panel). *C*, L-arginine-induced Ca^2+^ responses of two ORNs of an individual slice of the lateral MOE (red and purple trace). Application of SAG (200 µM; 10 s, middle panel; 50 s, right-hand-panel) triggered Ca^2+^ increases in both ORNs. *D*, L-arginine-induced Ca^2+^ responses of two ORNs of an individual slice of the lateral MOE (black and grey trace). Both ORNs did not respond upon application of AA (50 µM; 10 s, middle panel; 60 s, right-hand-panel) *E*, mean responses (±SEM), expressed as percentage of control response to L-arginine, of 14 ORNs (4 slices) in standard (dark grey columns) and bath solution with Xestospongin C 500 nM (green columns; respectively 5, 10 and 15 min after application of Xestospongin C; washout time was 5 min). No significant reduction of the Ca^2+^ responses was observed (p>0.05; paired Student's t-test). *F*, mean response amplitudes (±SEM) upon application of L-arginine (dark grey columns) and SAG (purple columns; 200 and 500 µM; 7 ORNs, 2 slices and 9 ORNs, 2 slices, respectively). The response amplitudes to SAG were lower but not significanty different from the L-arginine responses (p>0.05; paired Student's t-test). [AA, arachidonic acid].

We next examined whether phosphatidylinositol-3-phosphate (IP_3_), one of the PLC-mediated hydrolysis products of phosphatidylinositol-4,5-bisphosphate (PIP_2_), is involved in amino acid signaling. Application of Xestospongin C, a membrane-permeant blocker of IP_3_-mediated Ca^2+^ release from intracellular stores, had no detectable effect on L-arginine responses (14 ORNs, 4 slices; Figure 4A, B and E), indicating that in *Xenopus laevis* the IP_3_ receptor does not participate in amino acid signaling. We therefore hypothesized that DAG, the second hydrolysis product of PIP_2_, could possibly participate in the transduction cascade of the lateral subpopulation of amino acid-sensitive ORNs. Indeed, application of the DAG analogue SAG, triggered Ca^2+^ increases in the majority (66%) of L-arginine-sensitive ORNs in the lateral MOE (16 out of 24 ORNs, 4 slices; Figure 4C and F). On the other hand, application of arachidonic acid, a metabolite of DAG, did not affect amino acid-sensitive ORNs (18 ORNs, 8 slices; Figure 4D).

### Putative TRP channel blockers reduce amino acid responses in the lateral MOE

**Figure 5:**
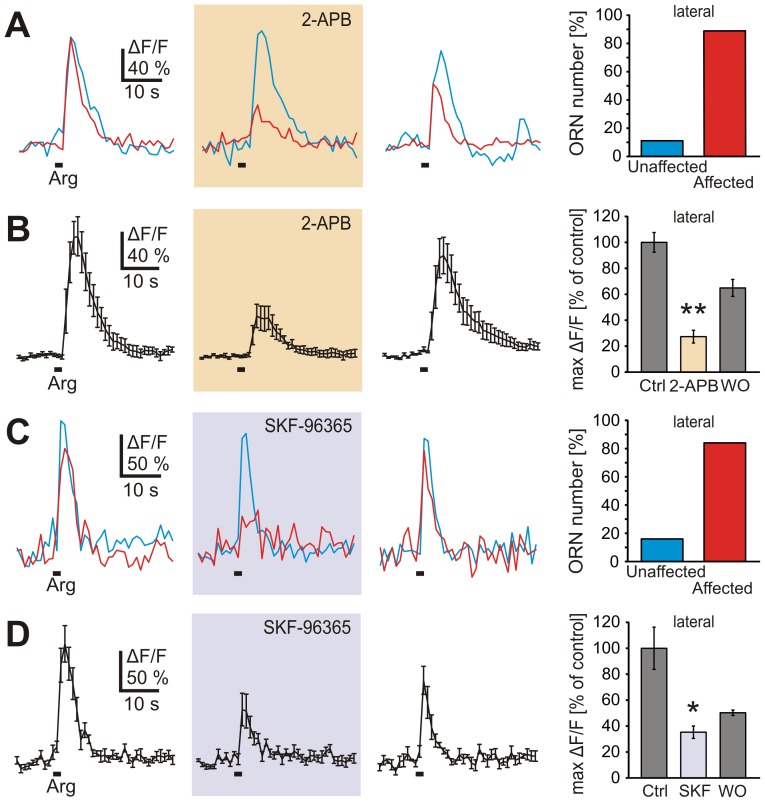
TRP channel blockers affect most of the L-arginine-induced responses. *A*, L-arginine-induced Ca^2+^ responses of two ORNs of an individual slice of the lateral MOE (cyan and red trace). Incubation with 2-APB (100 µM; application for 5 min; orange-shaded panel) led to a strong reduction of the Ca^2+^ transient in one of the ORNs (red trace), whereas the second ORN was not affected (cyan trace). The response of the affected ORN recovered after 10 min of washout (right-hand traces). The bar diagram shows ORNs unaffected and affected by 2-APB (100 µM; 5 min), expressed as percentage of total ORNs tested (27 cells, 6 slices). *B*, mean Ca^2+^ transient (±SEM) of seven L-arginine-responsive ORNs of an acute slice preparation (lateral MOE; only blocker-affected ORNs included). With 2-APB in the bath solution (100 µM; 5 min; orange-shaded panel) the mean response is partially suppressed. After 10 min washout the response recovered almost completely (right-hand trace). The bar diagram shows that application of 2-APB (100 µM; 5 min) reduced the amplitude of the Ca^2+^ transients to 27% of the control application (24 ORNs, 6 slices). *C*, L-arginine-induced Ca^2+^ responses of two ORNs of another individual slice of the lateral MOE (cyan and red trace). Incubation with SKF-96365 (80 µM; 10 min; lilac-shaded panel) led to a strong reduction of the Ca^2+^ transient in only one of the two ORNs (red trace). The response of the affected ORN recovered after 10 min of washout (right-hand traces). ORNs unaffected and affected by SKF-96365, expressed as percentage of total ORNs tested (histogram; 25 cells, 4 slices). *D*, mean Ca^2+^ transient (±SEM) of four L-arginine-responsive ORNs of an acute slice preparation (lateral MOE; only blocker-affected ORNs included). With SKF-96365 in the bath solution (80 µM; 10 min; lilac-shaded panel) the mean response is partially suppressed. After 10 min washout the response slightly recovered (right-hand trace). Application of SKF-96365 (80 µM; 10 min) reduced the amplitude of the Ca^2+^ transients to 35% of the control application (bar diagram; 21 ORNs, 4 slices). Statistical analysis was performed using paired Student's t-test; *p<0.05, **p<0.01. [SKF, SKF-96365].

In VRNs of mouse DAG has been shown to directly gate TRPC2 channels [17]. As specific TRPC2 inhibitors are not available, we investigated the effect of two putative TRP channel blockers, 2-APB and SKF-96365, that have been reported to affect TRPC2-mediated responses in the rodent VNO [17], [18]. We found that 24 out of 27 L-arginine-sensitive ORNs in the lateral MOE (6 slices), i.e., 89%, were reversibly affected by 2-APB (Figure 5A). The remaining three responsive ORNs were not affected at all. On average, L-arginine-induced Ca^2+^ transients of the affected ORN pool showed 73% reduction under 2-APB (Figure 5B). The second blocker, SKF-96365, gave similar results. SKF-96365 reversibly reduced L-arginine-induced Ca^2+^ transients in 21 out of 25 responsive ORNs (4 slices), i.e., in 84% of the ORNs. The remaining four responsive ORNs were not affected at all. On average, L-arginine-induced Ca^2+^ transients of the affected ORNs showed 65% reduction under SKF-96365 (Figure 5C, D).

## Discussion

In the present study, we investigated the transduction pathway of the amino acid-sensitive ORN subpopulation of the *Xenopus* lateral MOE. Using a pharmacological approach, we find most amino acid responses in this ORN subpopulation to be PLC-dependent. The presence of some PLC-independent amino acid-sensitive ORNs may reflect the presence of a few elements of the medial olfactory stream, which is not completely segregated from the lateral stream at the level of the MOE [6]. Consistent with this interpretation, the percentage of PLC-independent amino acid responses is much higher in the non-lateral regions of the MOE, where a preponderance of the medial stream can be expected [6]. PLC-independent amino acid responses appear to be heterogeneous, some of them forskolin-sensitive [12], but others forskolin-insensitive (this study). Thus, amino acid responses in the MOE of *Xenopus laevis* may be mediated via three different pathways: the main one, preponderant in the lateral MOE, depends on PLC, another, enriched in non-lateral MOE, employs cAMP, and a third pathway requires neither PLC nor cAMP. This heterogeneity in signaling pathways for amino acid responses is reminiscent of results obtained for mudpuppy, another amphibian [19]. The involvement of more than one neuronal population and signaling pathways appears to be an evolutionary ancient feature of neuronal representation of amino acids, since a contribution of both ciliated and microvillous ORNs has been suggested for several teleost fish species [20]–[24].

The PLC-mediated hydrolysis of PIP_2_ generates IP_3_ and DAG. The latter is then further metabolized to various polyunsaturated fatty acids, e.g., arachidonic acid, by the activity of the enzyme DAG lipase (for a review see [25]). All three metabolites have been reported to participate in the transduction of pheromonal stimuli in vertebrate VRNs (IP_3_: [14], [26]; DAG: [17], [18]; arachidonic acid: [16], [18]). The results of the present study suggest that only one of these pathways, DAG-mediated Ca^2+^ influx, is used for transmission of amino acid responses in the MOE of *Xenopus*. Neither the IP_3_ receptor antagonist Xestospongin C, nor application of arachidonic acid affected amino acid-responsive ORNs in the lateral MOE. On the other hand, the application of the DAG analogue SAG induced Ca^2+^ increases in the majority of amino acid-sensitive ORNs of the lateral MOE. These data suggest the transduction pathway in the MOE of larval *Xenopus* to be similar to that of vomeronasal sensory neurons. In VRNs of mouse a direct link between pheromone-induced PLC activity, production of DAG, and DAG-dependent gating of TRPC2 channels has been established [17], [25]. We show here that two putative TRP channel blockers (2-APB and SKF-96365) reversibly attenuate amino acid responses in the lateral MOE. Despite lack of specificity [27], [28], these blockers constitute the best available means to assess involvement of TRP channels using a pharmacological approach. Indeed, 2-APB and SKF-96365 have been shown to affect TRPC2-mediated responses in the rodent VNO [17], [18]. We obtained almost identical results for both blockers with respect to the percentage of affected cells and the amount of response reduction. This percentage is also very similar to that found for ORNs affected by the PLC inhibitor U-73122, suggesting that PLC-dependent and TRP channel blocker-sensitive ORNs may constitute the same subpopulation. Furthermore, the presence of some L-arginine-sensitive ORNs in our experiments that are not affected by the TRP channel blockers excludes an unspecific general effect on neuronal activation. Further investigation will be necessary to substantiate the involvement of TRP channels in the amino acid response pathway examined here.

Our results strengthen the importance of the *Xenopus laevis* olfactory system as a functional intermediate between aquatic and terrestrial vertebrates. The vomeronasal organ of *Xenopus* already closely resembles that of rodents [1], [4], [6], [7], [29]. On the other hand, the MOE of *Xenopus* is still very similar to the single sensory surface in teleost fishes [5]. It contains ciliated as well as microvillous ORNs [4], expresses *v2r* genes [7], has at least two [12] and possibly three (our current results) diverse transduction mechanisms, and features spatially segregated olfactory streams with distinct molecular and cellular mechanisms [6]. Finally, our data show that microvillous receptor neurons endowed with a PLC-dependent transduction and V2Rs appear to be evolutionary conserved among vertebrates, and are possibly related to detection of non-volatile molecules. In most mammals microvillous neurons expressing V2Rs are located in the VNO, are specialized for the detection of pheromones ([30]; but see [31]), and employ a PLC and DAG-mediated transduction pathway [17], [25]. In teleost fishes the same cell type is present in the unique sensory surface, where it detects amino acids via V2Rs [32] and signal via PLC [24]. In the fully aquatic larvae of *Xenopus* studied here, microvillous ORNs located in a lateral olfactory processing stream also detect amino acids [6] and signals via PLC (our current results), further supporting the hypothesis that amphibian V2Rs might be amino acid receptors like their teleost counterparts [33]–[35]. During metamorphosis, a third olfactory epithelium arises in the so-called middle cavity (water-filled), and the larval MOE is reorganized into the principal cavity, which becomes air-filled. Thus one might expect amino acid responses, *v2r* gene expression, and PLC-mediated signalling to migrate from the larval MOE to the adult middle cavity. It will be interesting to see whether this combination of microvillous ORNs, PLC-dependent signal transduction and *v2r* expression holds up in the tripartite olfactory organ of adult *Xenopus laevis* [36].
